# Stress, health and quality of life of female migrant domestic workers in Singapore: a cross-sectional study

**DOI:** 10.1186/s12905-017-0442-7

**Published:** 2017-10-10

**Authors:** S. G. Anjara, L. B. Nellums, C. Bonetto, T. Van Bortel

**Affiliations:** 1 Cambridge Institute of Public Health, University of Cambridge School of Clinical Medicine, Box 113 Cambridge Biomedical Campus, Forvie Site, Robinson Way, Cambridge, CB2 0SR UK; 2 Section of Infectious Diseases and Immunity, Department of Medicine, Imperial College London, 8th Floor Commonwealth Building, Hammersmith Hospital, Du Cane Road, London, W12 0NN UK; 3 Department of Neurosciences, Biomedicine and Movement Sciences, Section of Psychiatry, University of Verona, P.le L.A. Scuro 10, 37134 Verona, Italy; 4 Institute for Health and Human Development, University of East London, Stratford Campus, Suite 250, University House, The Green, Water Lane, London, E15 4LZ UK

## Abstract

**Background:**

There is a global increase in migrant workers. In Singapore, there are over 230,000 migrant domestic workers (MDWs). Female MDWs may experience high levels of stress and social isolation, which may negatively impact on their health and quality of life. There have also been documented cases of abuse and exploitation. However, there is a lack of empirical research with this population. This study aimed to investigate factors impacting on the health and quality of life of female MDWs in Singapore, including socio-demographic and job related characteristics, stress, social isolation, and working management style.

**Methods:**

A cross-sectional survey was carried out with 182 female MDWs in Singapore. The survey examined health and quality of life (WHOQoL-Bréf), social connectedness (the Friendship Scale), and preferred and experienced working management style (the Theory X and Theory Y Questionnaire). Descriptive analyses were carried out in addition to ANOVA, t-tests, and chi-square tests, followed by a multivariate analysis using linear regression.

**Results:**

Participants were found to have good overall quality of life and satisfaction with health. Age and working experience were found to be significantly (*p* < 0.05) associated with overall quality of life and three domains (psychological, social, and environmental health). Agreement between experienced and preferred working management style was also found to be associated with higher quality of life scores (with the exception of the social relationships domain). Though women reported relatively good overall quality of life, more than half of participants reported feeling stressed. In addition, nearly 20% of participants reported being isolated or very isolated. Stress was identified to be associated with isolation. In the multivariate analysis, stress was found to contribute to worse quality of life in all domains except social relationships, after adjusting for confounders. Social connectedness was positively associated with all domains of quality of life, and agreement of working management style was positively associated with physical health, psychological health and environmental quality of life.

**Conclusions:**

The findings serve as an evidence-base pointing to the need for policies aimed at decreasing stress and social isolation among female MDWs in order to improve their health and quality of life.

**Electronic supplementary material:**

The online version of this article (10.1186/s12905-017-0442-7) contains supplementary material, which is available to authorized users.

## Background

There is a global increase in the number of migrant workers, both skilled and unskilled. Singapore is one of Asia’s most prosperous countries, with similar per capita Gross Domestic Product (GDP) to that of leading nations in Western Europe [1]. In 2012, unemployment rates among Singaporean citizens and permanent residents were at a low of 2.8% [2]. In view of its small population of 5 million, declining birth rates (7.7 per 1000 population in 2012), ageing populace and rising cost of living, Singapore imports migrant workers to reduce labour shortages [3, 4]. Transient labour forces from neighbouring countries are lured by the higher wages they can earn in Singapore [5, 6].

According to the Asian code of piety, women should care for children and older adults, in addition to being responsible for housekeeping [7, 8]. However, over the last four decades, Singapore has experienced an increase in the number of women entering the workforce, as dual income has become necessary to meet the rising cost of living [9]. This reflects a global pattern producing a ‘global care chain’ [10, 11] in which a cyclical demand for domestic workers is created as women enter the work force, and women migrate to fill their domestic roles. This cycle both contributes to development in receiving countries, and deficits in domestic roles (e.g. carers of ‘left behind families’) in sending countries [12].

With an increasing demand for domestic workers, in 1978 the Singaporean government introduced the Foreign Domestic Worker Scheme to meet this need. By 2015, there were over 230,000 migrant domestic workers (MDWs) in Singapore compared with 20,000 in 1987 [13, 14]. While the state has acknowledged the need for domestic help, there are numerous restrictions on MDWs as a condition of their work permits. For example, MDWs are prohibited from settling permanently in Singapore [4]. Regulatory measures introduced by the state include *inter-alia* a two-year working contract, minimum age of 18 (raised to 23 on 1 January 2005), and the prohibition of pregnancy with or marriage to Singaporeans [15]. Hitherto, family members are prohibited from joining MDWs in Singapore. This has detrimental effects not only on the overall health and quality of life of MDWs, but also on their families left-behind [16], and there is a body of literature documenting the mental and physical effects of occupational stressors, as well as exposure to exploitation and abuse, among labour migrants [17–20].

MDWs also often face arbitrary rules not enforceable under law. They are bound by the contracts set by their individual employers who also sponsor their work permits. Although workers have the option to reject the terms of employment, there is pressure to accept these terms in order to retain their employment contracts and work permits, and be able to remit money home. MDWs are also excluded from the Employment Act, the key legislation that covers employment conditions for workers in Singapore [15]. For example, MDWs often work around the clock, as there are no stipulated working hours. Bullying or abuse also often occurs, as workers are required to live with their employers [4]. They are deterred from filing a complaint when abused as their work permits may be cancelled. This makes them vulnerable to high levels of stress, mental and physical strain, social isolation and a lower quality of life.

In 2014, a Burmese female MDW broke both legs while escaping from her employer’s high-rise condominium, causing a strain on Burma-Singapore relations and leading to a ban of Burmese workers going to Singapore [21–23]. Exploitation and abuse among MDWs, and the strain in bilateral relationships resulting from cases of this, are not limited to Singapore and its labour suppliers. For example, there are reports that MDWs in the Arab states face sexual abuse, harassment, and 21-h days. The Kefala system in the Arab states ties MDWs to their employers in a visa sponsorship system much like in Singapore. This means that MDWs are unable to switch jobs without their employer’s permission [24, 25].

While the Singapore government has mandated a weekly day off with effect from 1 January 2013 [15], workers may negotiate exchanging their days off for extra money. As some employers still believe that workers’ entitlement to weekly rest days (and their resulting freedom) may be problematic, and because of the profitability of increased work hours for employees [26], many employers arrange to compensate them in exchange for no days off.

Stress and mental health are inversely related [27]. The World Health Organization defines mental health as “a state of well-being in which the individual…can cope with the stresses of life” [28]. According to the *Demand-Control Theory* of stress, people working in high-strain jobs are more prone to a range of psychological and physical problems [29]. MDW’s roles are characterised by excessive demands and limited autonomy, which predispose a worker to psychological ill health. Although no previous research has investigated quality of life among MDWs, suicide figures shed some light on this [30]. Between 1999 and 2005, 147 MDWs were reported to have died from accident or suicide [31], and suicide is underreported and often recorded as another cause of death. Human Rights Watch reported that poor working conditions, anxiety over unpaid debts, social isolation and prolonged confinements were cited by workers’ peers as reasons for suicide [31].

The *Demand-Control Theory* of stress also acknowledges how social support can impact on psychosocial health [29]. Good social support has been found to protect against the detrimental effects of work demands, and higher levels of autonomy and social support have been linked to better quality of life [32]. Forced to live with their employers, MDWs may lack social support and positive relationships outside work, especially if they have no access to a weekly day off. An advocate of migrant workers’ rights stated: “Many 'no day off' employers do their utmost to cut off their workers from communication with the outside world. I am certain that it is damaging to their mental health, so that suicides and the infliction of harm on employers' family members should be seen in this context”. This suggests that stressful working conditions and a lack of social support may contribute to harmful behaviours to self or others.

Though MDWs may be at increased risk of poor mental health and quality of life, there is a lack of empirical research on this population. Most academic publications focus on migration economics and the social implications of MDWs’ presence in Singapore. However, it is becoming necessary to look at their general quality of life following the legalisation of a weekly day off for MDWs and the subsequent lobbying to place them under the Employment Act. This is especially salient as there are employers who doubt the benefits of a weekly day off [8]. While cases of MDW abuse reported in the media only comprise a small fraction of the population, this can be likened to the tip of an iceberg. The majority of workers do not receive attention in a typical ‘out of sight, out of mind’ paradigm. Understanding their socio-demographic characteristics, stress, social connectivity, health, and quality of life may assist policy makers in developing pro-health policies that are neither a reaction to bad press nor subject to voters’ demands. This is especially important as Singapore, Indonesia, and the Philippines, among others, entered into the ASEAN Economic Community on 1 January 2016, facilitating labour movement between member countries.

In addition, there is a global increase in attention towards migrant worker issues, in the context of migration, mental health in the workplace, social justice and human rights, as well as the global suicide agenda. Human Rights Watch in 2014 published a qualitative case study with 99 MDWs in the United Arab Emirates, eerily titled “I already bought you” expressing the sentiments of many Arab employers of MDWs [33]. Unfortunately, in Singapore, the sentiment is perhaps similar. Information gathered in this study contributes to the wider literature on migrant worker issues, providing the so-called population norm for the quality of life and social connectivity of MDWs in general, against which individual comparisons may be made.

## Methods

### Design, aims & setting

As the first empirical quantitative study into the health and quality of life of Female MDWs (FMDW) with a large respondent base representative of FMDWs in Singapore, this study aimed to investigate factors impacting on FMDWs’ health and quality of life (defined as “an individual’s perception of their position in life in the context of the culture and value systems in which they live and in relation to their goals, expectations, standards and concerns,” assessed in relation to satisfaction with health, and physical, psychological, social, and environmental domains [34]). The specific objectives were to use data from a cross-sectional survey of FMDWs in Singapore to: (1) describe FMDWs’ socio-demographic and job related characteristics, levels of stress, social connectedness, experienced and preferred working management style, and health and quality of life; (2) explore the relationship between health and quality of life and: socio-demographic and job related characteristics, stress, social connectedness, and working management style; (3) examine the relationship of socio-demographics, stress, social connectedness and working management style, with health and quality of life; and (4) consider the implications for policy and practice.

This cross-sectional survey was conducted in Singapore during April 2012 with a sample of FMDWs. Data on socio-demographics (age, level of education, marital status, country of origin, religion), job related characteristics (first job, previous work country, length of working experience), perceived stress, social connectedness (the Friendship Scale), preference for working management style and current working management situation (the Theory X and Theory Y Questionnaire), and health and quality of life (WHOQoL–Bréf) [35] were collected. The survey was conducted in English. Translation resources were available through the field researcher (SGA, who is multilingual) and the gatekeeping organisations, however all participants were found to have sufficient English proficiency to complete the questionnaire and provide fully informed consent.

Ethical approval for the study was obtained from the King’s College London Psychiatry, Nursing and Midwifery Research Ethics Committee on 3 April 2012 PNM/11/12–77. All participants provided full consent to taking part in the study.

This was a community-based study and not a health services study, and thus ethical approval was not needed from medical or health bodies in Singapore as this research was with community members and no patients or health services were involved.

### Participants & materials

Participants were female migrant domestic workers aged 18 or older who had access to days off with non-standard frequency, and held a Work Permit in Singapore, meeting the criteria for a ‘foreign domestic worker’ as stated by the Singapore government [15]. Proficiency in English and a minimum of 8 years of formal education are criteria for a Work Permit in Singapore, and thus all participants were found to have sufficient proficiency to understand and fully engage with the questionnaires.

The Friendship Scale is a short, user-friendly, standalone questionnaire which measures six dimensions of social connectivity, including: ease in relating to others; isolation from others; having someone to share with; ease to get in touch; feeling separate from others; and loneliness [36]. For each of the six items, five response options are given, ranging from “Not at all” to “Almost always”. Based on this structure, it is recommended that the total score is classified in quintiles, which are labelled “Very Isolated”, “Isolated”, “Some Isolation”, “Connected” and “Very Connected”. Internal consistency analysis of the Friendship Scale derived an average inter-item reliability (Cronbach’s α = 0.64).

Preference for working management style and current management style (authoritarian (X) or laid back (Y)) were assessed using the Theory X and Theory Y Questionnaire adapted for the study. Theory X and Theory Y are theories of human motivation and management developed by Douglas McGregor in the 1960s. Theory X highlights the importance of strict supervision and external rewards to motivate workers, while Theory Y stresses the motivating role of job satisfaction to encourage workers to perform [37].

The WHOQoL-Bréf is a self-report questionnaire which provides a measure of overall satisfaction with health and quality of life (QoL), as well as quality of life in the physical, psychological, social, and environmental domains [35]. The Australian version of the questionnaire was used, and participants’ responses were scored using the Australian Scoring Algorithm. When more than 20% of responses per domain are missing, the algorithm scores only the domains for which complete data are available. Overall quality of life and satisfaction with health were scored from 1 to 5, and mean scores were calculated. Mean scores were also calculated for the four domains of quality of life (physical, psychological, social, and environmental) and converted to a 0–100 scale as suggested by the manual, based on the highest possible raw scores, for easy interpretation and comparisons with other validated instruments or published community norms. Internal consistency analysis of the WHOQoL-Bréf derived a high inter-item reliability (Cronbach’s α = 0.89).

### Data collection

The field researcher approached a number of civil society organisations for information on MDW groups in Singapore. Both the Indonesians and the Filipinos have an umbrella organisation that is organised among themselves: the Indonesian Family Network (IFN) and the Filipino Family Network (FFN). Through Transient Workers Count Too (TWC2), a non-profit organisation focusing on advocacy for the rights of migrant workers, the researcher contacted the leaders of both FFN and IFN. Subsequently, meetings were arranged in public places of gathering, where MDWs usually congregate during their days off. Leaders of the MDW groups connected potential participants to the researcher. The researcher introduced the research project and provided research information sheets, as well as contact information (e.g. in case they should choose to participate at a later date or have any further queries). Potential participants were also invited to ask questions. The researcher ensured that women felt they had sufficient English language proficiency both to fully engage with the questionnaire and to provide fully informed consent.

After consenting to participate, women were provided questionnaires which took approximately half an hour to complete. The questionnaires were anonymous and did not elicit personal identifying information, ensuring that data could not be traced back to individual participants.

Participants were recruited using convenience sampling. In order to guide recruitment, we conducted a power analysis based on the reported population norms and effect sizes for the WHOQoL-Bréf. The general norm for the social relationships quality of life domain is 71.5 (SD = 18.2), which we selected to use for our sample size calculation given the particular focus of the research on social isolation. We hypothesised that the social relationship score for our sample would be lower than the general norm, and calculated that we would have 80% power at the 5% significance level to detect whether the sample had a social relationship domain score of 65 when the sample size was 62.

A total of 182 out of 220 women given questionnaires participated in the study (response rate = 82.7%). It was noted that some individuals declined to complete the questionnaires after looking at the WHOQoL-Bréf item on satisfaction with sexual relationships.

### Statistical analysis

Data were described by using mean (sd) or n (%) depending on variable type. The relationship between continuous variables and categorical characteristics were tested by ANOVA or t-test. The association between two categorical variables was tested by chi-square. All tests were bilateral at *p* < 0.05. No multiple comparison correction was applied to these explorative analyses. To examine the relationship of socio-demographics, stress, social connectedness and working management style with health and quality of life, a series of multiple block-stratified linear regression analyses were performed with QoL domains as dependent variables and socio-demographics (only those which were significantly associated with QoL at *p* < 0.05), stress (Yes vs No), social connectedness (Friendship Scale score) and agreement between experienced and preferred working management style (Yes vs No) as independent variables. Working experience length was not included in the models because of its collinearity with age. The statistical software used was SPSS 22 for Windows.

## Results

### Socio-demographic and job related characteristics

Participants reported a mean age of 35.1 years (sd 7.6, range 20–63). The majority of participants were from the Philippines (57.1%), while the second largest group was from Indonesia (37.4%). Only 39% had a high level of education (diploma/university). 43.6% of participants were single and 41.4% were married. Catholics and other Christians made up almost two thirds of the respondents (64.3%), while 30.8% were Muslims. Work experience ranged from 1 month to 396 months (mean 89.9, sd 72.9). A little more than half of the participants (52.7%) reported they were currently working in their first job. Those who have worked before had experience in their home country (33.7%), Arabian Peninsula (23.2%), Hong Kong/Taiwan (19.8%), Malaysia/Brunei (10.5%), or a previous employer in Singapore (11.6%) (See Table 1).

**Table 1:** Socio-demographic and job related characteristics of participants

Characteristics	Participants (*n* = 182)
Age (2 missing)	
< 30	50 (27.8%)
30–39	83 (46.1%)
40–49	38 (21.1%)
50+	9 (5.0%)
Education (5 missing)	
Low (Primary/Secondary)	108 (61.0%)
High (Diploma/University)	69 (39.0%)
Marital status (1 missing)	
Married	75 (41.4%)
Single	79 (43.6%)
Widowed/Divorced	27 (14.9%)
Country of origin	
The Philippines	104 (57.1%)
Indonesia	68 (37.4%)
Myanmar/Sri Lanka	10 (5.5%)
Religion	
Islam	56 (30.8%)
Catholic	76 (41.8%)
Christian	41 (22.5%)
Buddhist/Sikh	7 (3.8%)
No religion	2 (1.1%)
First job	
No	86 (47.3%)
Yes	96 (52.7%)
Previous work country (for women who worked before)	
Singapore	10 (11.6%)
Malaysia/Brunei	9 (10.5%)
Hong Kong/Taiwan	17 (19.8%)
Arabian Peninsula	20 (23.2%)
North America	1 (1.2%)
Home country	29 (33.7%)
Working experience (years) (5 missing)	
≤ 2	24 (13.6%)
2–6	71 (40.1%)
6–10	36 (20.3%)
10–20	41 (23.2%)
> 20	5 (2.8%)

This table displays the characteristics of the study participants (*n* = 182), including their socio-demographic characteristics and employment characteristics

### Quality of life, stress, social connectedness and working management style levels

The majority (73.6%) of participants showed an overall good or very good quality of life, while only 5.4% declared a poor or very poor one. Regarding satisfaction with health, almost 80% of subjects were satisfied or very satisfied; only 3.9% were fairly dissatisfied or very dissatisfied. Over half of the respondents (52.5%) reported feeling stressed. Scores on the Friendship Scale ranged from 7 to 24, with a mean of 18.5 (sd 3.8). About 20% of participants were categorised as ‘Isolated’ or ‘Very Isolated’, while a little more than half (52.4%) of participants categorised as ‘Socially Connected’ or ‘Very Connected’. Regarding management style, 55.5% declared that they experience Theory Y leadership style (Generally Y or Strongly Y), while 6.4% had an autocratic leadership style. Looking at the preference for management style, about 75% preferred Y-theory management and only 4.7% an autocratic style. More than 60% of women declared an agreement between experienced and preferred management style (See Table 2).

**Table 2:** Quality of life, stress, social connectedness, and working management style levels

Instrument	Participants (*n* = 182)
	mean (sd), or n (%)
WHOQoL-Bréf	
Overall QoL	3.8 (0.7)
Satisfaction with health (1 missing)	4.0 (0.8)
I Physical health (3 missing)	67.7 (11.9)
II Psychological health (2 missing)	67.8 (12.0)
III Social relationships (11 missing)	63.6 (14.6)
IV Environment (3 missing)	66.2 (15.0)
Do you feel stressed? (20 missing)	
Yes	85 (52.5%)
No	77 (47.5%)
Friendship Scale (10 missing), quintiles	
Very isolated	9 (5.2%)
Isolated	25 (14.5%)
Some isolation	48 (27.9%)
Socially connected	51 (29.7%)
Very connected	39 (22.7%)
X-Y Theory Questionnaire	
Situation and management style (9 missing)	
0–15 Strongly X (Autocratic leadership)	11 (6.4%)
16–44 Generally X	66 (38.2%)
45–59 Generally Y	47 (27.2%)
60–75 Strongly Y (Effective leadership)	49 (28.3%)
Preference for management style (12 missing)	
0–15 Strongly X (Autocratic leadership)	8 (4.7%)
16–44 Generally X	35 (20.6%)
45–59 Generally Y	54 (31.8%)
60–75 Strongly Y (Effective leadership)	73 (42.9%)
Agreement between experienced and preferred management style (13 missing)	
Yes	107 (63.3%)
No	62 (36.7%)

This table describes the quality of life, stress, social connectedness, and working management style levels of study participants

### Relationship between quality of life and socio-demographic and job related characteristics

As shown in Table 3, age was found to be significantly (*p* < 0.05) associated with overall quality of life and three domains (psychological, social, and environmental health). Interestingly, all these aspects showed a positive trend with age (becoming older ameliorated satisfaction with life), with the exception of social relationships, which slightly decreased in the last category (50+). Women from Indonesia were suggested to have higher levels of overall quality of life, satisfaction with health, and environmental quality of life, whilst women from Myanmar and Sri Lanka had lower psychological quality of life scores. Years of working experience had the same pattern as age, with the last category (>20 years) showing the highest scores.

**Table 3:** Relationship between quality of life and socio-demographic and job related characteristics*

WHOQoL-Bréf, mean (sd)	Characteristics					*p*-value ANOVA
	Age					
	<30	30–39	40–49	50+		
Overall QoL (2 missing)	3.5 (0.9)	3.9 (0.5)	3.9 (0.8)	4.3 (0.7)		<0.001
Satisfaction with health (3 missing)	3.9 (1.0)	4.0 (0.7)	4.1 (0.6)	3.9 (1.3)		0.621
I Physical health (5 missing)	65.8 (13.1)	67.1 (12.0)	70.8 (10.1)	72.2 (9.7)		0.152
II Psychological health (4 missing)	62.4 (13.7)	68.4 (9.7)	71.9 (11.4)	78.2 (11.9)		<0.001
III Social relationships (13 missing)	59.4 (13.7)	62.9 (14.0)	69.4 (14.0)	66.7 (21.6)		0.017
IV Environment (5 missing)	61.2 (17.1)	65.6 (13.5)	70.3 (13.5)	79.2 (7.8)		0.001
	Country of origin					
	The Philippines	Indonesia	Myanmar/ Sri Lanka			
Overall QoL	3.7 (0.7)	4.0 (0.7)	3.3 (0.9)			0.002
Satisfaction with health (1 missing)	3.9 (0.7)	4.3 (0.7)	3.3 (1.3)			<0.001
IPhysical health (3 missing)	67.2 (12.7)	68.2 (10.3)	68.6 (14.6)			0.843
II Psychological health (2 missing)	68.4 (11.3)	68.4 (11.9)	57.9 (16.8)			0.027
III Social relationships (11 missing)	62.1 (14.3)	66.0 (13.9)	62.5 (22.3)			0.248
IV Environment (3 missing)	63.9 (14.5)	70.5 (12.5)	59.7 (25.6)			0.006
	Working experience (years)					
	≤2	2–6	6–10	10–20	>20	
Overall QoL (5 missing)	3.6 (0.8)	3.5 (0.8)	4.0 (0.4)	4.0 (0.6)	4.4 (0.9)	<0.001
Satisfaction with health (6 missing)	3.8 (0.9)	3.9 (0.9)	4.2 (0.6)	4.0 (0.8)	4.4 (0.6)	0.257
I Physical health (8 missing)	65.7 (13.9)	66.7 (12.5)	67.9 (10.6)	68.7 (11.4)	76.4 (4.07)	0.405
II Psychological health (7 missing)	64.8 (11.4)	65.4 (11.5)	69.2 (11.8)	70.6 (11.7)	78.3 (18.0)	0.028
III Social relationships (16 missing)	57.5 (14.4)	62.9 (14.3)	64.1 (13.9)	66.0 (14.7)	81.7 (9.1)	0.012
IV Environment (8 missing)	58.4 (17.7)	63.5 (14.1)	69.1 (12.5)	70.1 (14.5)	83.7 (8.7)	<0.001

*Only significant associations are reported, *p* < 0.05

This table describes the relationship between quality of life and socio-demographic and job related characteristics among study participants

In the exploratory analyses we also identified that stress was significantly associated with country of origin and religion. Age and years of working experience were found to be significantly associated with social connectedness. Following the same pattern as quality of life, this aspect showed a positive trend with both age and years of working experience (becoming older or having a longer working experience) ameliorating social connectedness (See Additional file 1: Table S1).

Interestingly, age and working experience showed the same pattern with experienced management style (older women or those with more working experience had a less authoritative leadership style). This trend was also evident in the relationship between age and preferred management style. Preferred management style was also found to be associated with marital status and country of origin. No significant association was found for agreement between experienced and preferred management style.

### Association between quality of life and stress, social connectedness and working management style

Not feeling stressed was found to be significantly associated with having a higher quality of life across all domains, and with being more socially connected. Social connectedness was also found to be significantly associated with higher quality of life scores in all domains with the exception of satisfaction with health, showing a positive trend. Agreement between experienced and preferred working management style was found to be significantly associated with a higher quality of life, with the exception of social relationships (See Table 4).

**Table 4:** Association between quality of life and stress, social connectedness, and working management style*

	Do you feel stressed?					*p*-value t test
WHOQoL-Bref, mean (sd)	No	Yes				
Overall QoL (20 missing)	4.1 (0.7)	3.6 (0.7)				<0.001
Satisfaction with health (20 missing)	4.3 (0.7)	3.8 (0.9)				<0.001
I Physical health (21 missing)	71.2 (12.0)	64.7 (11.4)				<0.001
II Psychological health (21 missing)	71.7 (12.1)	64.7 (11.4)				<0.001
III Social relationships (25 missing)	67.4 (13.0)	60.9 (14.7)				0.004
IV Environment (22 missing)	71.6 (14.0)	61.6 (14.6)				<0.001
	Do you feel stressed?					*p*-value Chi-Square or t-test
Friendship Scale, n (%) (24 missing)	No	Yes				
Very isolated/Isolated	7 (25.0%)	21 (75.0%)				0.001^a^
Some isolation	17 (37.8%)	28 (62.2%)				
Socially connected/Very connected	52 (61.2%)	33 (38.8%)				
Mean score (sd)	20.0 (6.5)	17.6 (3.4)				<0.001
	Social connectedness					*p*-value ANOVA
WHOQoL-Bref, mean (sd)	Very isolated	Isolated	Some isolation	Socially connected	Very connected	
Overall QoL (5 missing)	3.6 (0.9)	3.5 (0.8)	3.7 (0.7)	4.0 (0.6)	4.1 (0.7)	0.002
Satisfaction with health (6 missing)	3.6 (1.2)	3.8 (0.7)	4.0 (0.8)	4.0 (0.8)	4.2 (0.8)	0.156
I Physical health (8 missing)	58.7 (11.0)	64.9 (14.0)	65.7 (11.6)	68.3 (11.3)	72.8 (11.0)	0.005
II Psychological health (7 missing)	61.1 (10.8)	61.5 (12.9)	67.2 (9.1)	68.6 (11.9)	73.0 (13.7)	0.002
III Social relationships (16 missing)	52.8 (18.6)	58.3 (13.0)	60.9 (15.3)	63.3 (13.5)	73.6 (10.6)	<0.001
IV Environment (8 missing)	53.8 (17.4)	58.2 (16.9)	62.5 (13.2)	68.1 (13.0)	75.4 (12.5)	<0.001
	Agreement between experienced and preferred working management style					*p*-value t test
WHOQoL-Bref, mean (sd)	No	Yes				
Overall QoL (13 missing)	3.7 (0.8)	3.9 (0.7)				0.026
Satisfaction with health (13 missing)	3.8 (0.9)	4.1 (0.8)				0.027
I Physical health (13 missing)	64.6 (12.9)	69.6 (11.4)				0.009
II Psychological health (13 missing)	65.0 (12.7)	69.8 (11.5)				0.014
III Social relationships (18 missing)	61.2 (15.7)	65.0 (14.1)				0.107
IV Environment (14 missing)	61.4 (16.9)	69.1 (13.1)				0.001

*Only significant associations are reported, *p* < 0.05

^a^The categories ‘Very isolated’ and ‘Isolated’ were summed up due to low frequencies; the categories ‘Socially connected’ and ‘Very connected’ were summed up due to low frequencies

This table describes the association between quality of life and stress, social connectedness, and working management style

### Relationship between quality of life and socio-demographic characteristics, stress, social connectedness and agreement between experienced and preferred working management style

Table 5 presents the results of a series of regression models, with each QoL domain as a dependent variable. As shown, the model estimated for environment explains 33.0% of the variance of QoL. The models for overall and psychological health explain 21.8% and 20.8%, respectively, while the adjusted R-squares for physical health, social relationships and satisfaction with health indicate that these models do not explain much of the variance of quality of life (13.8%, 12.%9 and 12.8%, respectively).

**Table 5:** Linear regression models for quality of life with socio-demographic characteristics, stress, social isolation, and agreement between experienced and preferred working management style (*n* = 182)

	Dependent variable (Beta coefficients, p-value)					
Independent variables	Overall QoL	Satisfaction with health	Physical health	Psychological health	Social relationships	Environment
Block 1: Socio-demographics	Adj-R^2^ = 13.2%	Adj-R^2^ = 5.8%	Adj-R^2^ = 1.8%	Adj-R^2^ = 8.5%	Adj-R^2^ = 4.2%	Adj-R^2^ = 15.7%
Age (years)	0.216 (p = 0.009)	−0.029 (*p* = 0.738)	0.047 (*p* = 0.584)	0.207 (*p* = 0.013)	0.077 (*p* = 0.385)	0.170 (*p* = 0.027)
Country of origin (ref. Indonesia)	0.217 (*p* = 0.006)	0.174 (*p* = 0.036)	0.001 (*p* = 0.996)	−0.016 (*p* = 0.836)	0.103 (*p* = 0.218)	0.209 (p = 0.005)
Block 2: Stress	Adj-R^2^ = 7.1%	Adj-R^2^ = 5.6%	Adj-R^2^ = 5.6%	Adj-R^2^ = 7.8%	Adj-R^2^ = 3.4%	Adj-R^2^ = 6.4%
Do you feel stressed? (ref. Yes)	−0.250 (*p* = 0.002)	−0.243 (*p* = 0.005)	−0.197 (*p* = 0.021)	−0.236 (*p* = 0.004)	−0.110 (*p* = 0.205)	−0.167 (*p* = 0.029)
Block 3: Social isolation	Adj-R^2^ = 0.7%	Adj-R^2^ = 0.3%	Adj-R^2^ = 3.0%	Adj-R^2^ = 2.8%	Adj-R^2^ = 5.2%	Adj-R^2^ = 7.9%
Friendship Scale	0.120 (*p* = 0.158)	0.056 (*p* = 0.531)	0.201 (*p* = 0.025)	0.201 (*p* = 0.019)	0.277 (*p* = 0.003)	0.319 (*p* < 0.001)
Block 4: X-Y Theory Questionnaire	Adj-R^2^ = 0.8%	Adj-R^2^ = 1.1%	Adj-R^2^ = 3.4%	Adj-R^2^ = 1.7%	Adj-R^2^ = 0.1%	Adj-R^2^ = 3.0%
Agreement	0.115 (*p* = 0.118)	0.150 (*p* = 0.055)	0.203 (p = 0.009)	0.150 (*p* = 0.044)	0.062 (*p* = 0.428)	0.187 (*p* = 0.007)
% Variance Explained by the final model	21.8%	12.8%	13.8%	20.8%	12.9%	33.0%

This table presents the models in which linear regression was used to examine the impact of socio-demographic characteristics, stress, social isolation, and agreement between experienced and preferred working management style on quality of life for study participants

Older women showed higher overall quality of life, and psychological health and environmental quality of life. Country of origin was also found to be significant in some of the models, with women from Indonesia reporting higher overall quality of life, satisfaction with health and environmental quality of life. Stress explained 7.1% of the variance for quality of life and was significantly and inversely associated with all domains with the exception of social relationships (i.e. being stressed is associated with a lower quality of life). Social connectedness was another important determinant, as more socially connected women expressed higher quality of life levels in physical health, psychological health, social relationships and environmental quality of life. Women reporting an agreement between experienced and preferred working management style were also more likely to experience higher quality of life in physical health, psychological health and environmental quality of life.

## Discussion

The findings suggested that participants had a good level of satisfaction with overall quality of life and health. Interestingly, age and work experience were found to be positively associated with health and quality of life, with older women with more working experience reporting better quality of life across most domains. 63.3% of women reported agreement between experienced and preferred working management style, which was also associated with higher health and quality of life scores. More than half of participants reported feeling stressed, which was significantly associated with poorer quality of life.

Nearly 20% of participants reported being ‘Isolated’ or ‘Very Isolated’ despite having access to rest days, which is higher than the normative percentage for the Friendship Scale instrument [36]. Social support was found to be inversely related to stress, health, and quality of life, and MDWs who reported feeling stressed were more likely to be isolated. On the other hand, very socially connected women showed the highest quality of life scores. This may partly explain why older workers and those with more work experience had improved quality of life scores, as they are likely to have better social support.

The association between social connectivity and quality of life is in line with the Demand-Control Theory. This is also consistent with previous research demonstrating the relationship between social isolation and poor health outcomes, and the protective effects of social support [38–41], and reflects research suggesting that the six dimensions of the Friendship Scale (ease in relating with others; isolation from others; having someone to share with; ease to get in touch; feeling separate from others; and loneliness) are associated with self-esteem and the psychological and social domains of the WHOQoL-Bréf [36]. The salience of social isolation may be particularly significant for these migrant women due to the loss of social support through migration, which has also been shown to have a negative effect on psychological health and quality of life [42–49], and is consistent with previous research highlighting the significance of social isolation among migrants, and its relationship with poorer health outcomes [50–54].

A mismatch between pre-migration aspirations and the reality of work may increase stress and contribute to poorer quality of life among new MDWs [55]. On the contrary, older and more experienced workers could have developed more effective stress coping strategies. More experienced workers may also be more acculturated and have adjusted to the host country, resulting in fewer experiences of stressful situations. Accordingly, they might have formed better social support networks, and acquired access to social and health resources. Older and more experienced FMDWs may also have learned to manage expectations better, resulting in better quality of life. There is very limited data on the relationship between migration, age, time since migration, social support, and health among migrant domestic workers – particularly those who migrate without their families or other social support networks. Furthermore, the evidence is inconsistent, with research suggesting both that greater time since migration is not associated with increased social integration or access to social support resources [56], and that greater length of residency is associated with improved health outcomes [57].

It should be noted that the stress these communities experience is likely to be multidimensional, and lower quality of life may be caused not only by a single stressor but also the continuing effects of other life events or chronic stress [58]. MDWs may be exposed to a range of stressors including financial burden, separation from family, violence, exploitation, immigration challenges, culture shock, language barriers, limited agency and being overworked, which may contribute to poor quality of life, social isolation, and stress. This is highlighted in a recent survey of MDWs in Singapore, which identified that 24% of women had poor mental health, 74% experienced restriction of movement, 51% had been verbally abused, and 40% were given less than one rest day a week [59].

There may be additional factors which are contributing to these outcomes among MDWs. For example, the theory of selective migration argues that people with poorer social skills or fewer social networks may migrate for work rather than stay in their hometowns [60]. As a result, these workers may report lower ratings on the *Social Relationship* domain of quality of life. Further research should be conducted to examine these aspects.

Low-skilled migrant workers face difficulties in accessing health services in many countries, let alone mental health or social services, due to lack of entitlement/access, language barriers, strict working hours, and lack of knowledge of services available in host countries [4, 9, 31, 61]. Workers’ poorer quality of life can result in decreased productivity [32, 62–69], and may also result in other social consequences, for example antisocial or criminal behaviour [70–73], or poor health outcomes [64–69]. The current study highlights the social and health needs of this population, and is consistent with previous research elsewhere including Hong Kong and Spain, pointing to the stressors and risk of exploitation experienced by domestic workers [74, 75]. Our findings point to the urgent need for immigration and employment policies promoting workers’ well-being and targeting abuse and exploitation, in line with international priorities to improve conditions and health outcomes among workers globally, for example the World Health Organization’s Healthy Workplaces model for action [76].

Although this research is empirical and includes a large sample of MDWs, it is not representative of those without rest days or residing at NGO or Embassy Shelters (who often have had problems with their employers or work permits). As this study includes MDWs who may have relatively better circumstances associated with their allowance of rest days, it is anticipated that in a sample representative of all MDWs, the quality of life and social support ratings may be lower than in our findings. While the sample is limited, there were also advantages, including that it enabled qualitative data to be collected (reported elsewhere).

Additionally, the questionnaires were in English. While all participants were proficient in English, there are two important issues relating to the language of the research that should be considered. In any research in which the language of the researcher, the language of the research instruments, and the native language of the participants differ, there is the potential for the trustworthiness of the research to be affected [77]. Thus, while the participants were proficient in English and translation resources were available (though were not needed by any participants), language barriers may still have impacted on their interpretations of the questions and their responses, and consequently the data generated [78, 79]. The second issue relating to language is that the study may not be representative of MDWs who are less proficient in English, and thus who did not participate in the study, though they are likely to represent a minority of migrant domestic workers with Work Permits in Singapore given the language requirements [80]. These individuals may be more likely to be isolated, exploited, or experience barriers to accessing health or social resources, and thus experience poorer health or quality of life.

## Conclusion

This is the first empirical study on the quality of life of migrant domestic workers in Singapore, a cosmopolitan city-state with a large non-citizen population, which has subsequently been reinforced by similar research demonstrating the mental health needs of this population in light of abuse and exploitation [59]. Overall, participants were found to have good quality of life and satisfaction with health. However more than half of participants reported feeling stressed, and nearly 20% of participants experienced isolation, both of which were associated with poorer quality of life outcomes. With globalisation and the projected increase in the need for migrant workers in many countries, there is a need for policies (e.g. access to rest days) to support their health and quality of life, and facilitate their access to coping resources [59].

However, there remains a need for further research on the health and quality of life of MDWs, including those who may be more marginalised or isolated (e.g. those unable to take advantage of a rest day). This is underscored in a recent systematic review on the health of MDWs, in which only six papers were identified that included Singaporean domestic workers in their samples, two of which used the same data [81]. Future research should look more closely into the prevalence of mental disorders among MDWs in Singapore and their correlation with abuse, in order to inform future policy. It is also worthwhile to examine whether the ASEAN Human Rights Declaration has made a difference in protecting the interests of migrant workers in Singapore, many of whom come from ASEAN countries. Applied quality of life research and comparisons of policy and practice regarding MDWs in the region and globally are necessary to provide insight into the health and quality of life of MDWs, that of their left-behind families, and their resulting needs.

Although many workers reported good social connectivity, alarmingly – especially in light of this study with MDWs who do have access to a day off – nearly one fifth reported social isolation, which was associated with poorer quality of life. The findings contribute to an evidence-base which should inform the development and implementation of policies aimed at reducing women’s exposure to stressors (e.g. exploitation, or conditions that exacerbate women’s social isolation, for example long working hours or a lack of access to days off). If effective, such measures could ultimately positively impact on female migrant domestic workers’ health and quality of life, which is a major issue in the wider ASEAN region and indeed the world.

## Additional files

Additional file 1: Table S1. Stress, social connectedness, and working management style in relation to socio-demographic and job related characteristics. This table shows the relationship between socio-demographic and job related characteristics and stress, social connectedness, and working management style. (DOCX 38 kb)

## Abbreviations

FMDW: Female migrant domestic worker
MDW: Migrant domestic worker
QoL: Quality of life
