# Supplementary material for: Stress, health and quality of life of female migrant domestic workers in Singapore: a cross-sectional study
Source: BMC Womens Health. 2017 Oct 10;17:98. doi: 10.1186/s12905-017-0442-7 (PMC5634837; doi:10.1186/s12905-017-0442-7)
Supplement: Supplementary file 1 — Stress, social connectedness, and working management style in relation to socio-demographic and job related characteristics. This table shows the relationship between socio-demographic and job related characteristics and stress, social connectedness, and working management style. (DOCX 38 kb) [file 12905_2017_442_MOESM1_ESM.docx]

**Supplementary Table 1** Stress, social connectedness, and working management style in relation to socio-demographic and job related characteristics*

|  | **Characteristics** | | | | |  |
| --- | --- | --- | --- | --- | --- | --- |
| **Stress, n (%)** |  |  | | |  | **p-value**  **Chi-square** |
|  |  | **Country of origin** | | |  |  |
|  |  | **The Philippines** | **Indonesia** | **Myanmar/ Sri Lanka** |  |  |
| Do you feel stressed? (20 missing)  Yes  No |  | 59 (64.1%)  33 (35.9%) | 22 (35.5%)  40 (64.5%) | 4 (50.0%)  4 (50.0%) |  | 0.002 |
|  |  | **Religion** | | |  |  |
|  | **Islam** | **Catholic** | **Christian** | **Buddhist/ Sikh** | **No religion** |  |
| Do you feel stressed? (20 missing)  Yes  No | 18 (35.3%)  33 (64.7%) | 45 (67.2%)  22 (32.8%) | 19 (52.8%)  17 (47.2%) | 1 (16.7%)  5 (83.3%) | 2 (100.0%)  0 (0.0%) | 0.003** |
| **Friendship Scale, mean (sd)** |  |  |  | |  | **p-value ANOVA** |
|  |  |  | **Age** | |  |  |
|  |  | **<30** | **30-39** | **40-49** | **50+** |  |
| Total score (12 missing) |  | 16.6 (4.2) | 18.3 (3.2) | 20.8 (2.9) | 21.2 (2.6) | <0.001 |
|  |  | **Working experience (years)** | | |  |  |
|  | **≤2** | **2-6** | **6-10** | **10-20** | **>20** |  |
| Total score (15 missing) | 16.4 (3.3) | 17.9 (3.9) | 18.5 (3.3) | 19.9 (3.4) | 23.7 (0.5) | <0.001 |
| **X-Y Theory Questionnaire, mean (sd)** |  |  |  | |  | **p-value ANOVA** |
|  |  |  | **Age** | |  |  |
|  |  | **<30** | **30-39** | **40-49** | **50+** |  |
| Situation and management style  (11 missing) |  | 39.8 (19.8) | 45.9 (17.4) | 54.0 (15.7) | 65.6 (12.2) | <0.001 |
|  |  | **Working experience (years)** | | |  |  |
|  | **≤2** | **2-6** | **6-10** | **10-20** | **>20** |  |
| Situation and management style  (11 missing) | 40.2 (18.3) | 43.8 (18.4) | 48.7 (18.3) | 49.5 (18.1) | 67.4 (11.0) | 0.019 |
|  |  |  | **Age** | |  |  |
|  |  | **<30** | **30-39** | **40-49** | **50+** |  |
| Preference for management style  (14 missing) |  | 49.7 (18.2) | 52.8 (15.8) | 56.3 (16.6) | 65.8 (11.4) | 0.038 |
|  |  | **Marital status** | | |  |  |
|  |  | **Married** | **Single** | **Widowed/divorced** |  |  |
| Preference for management style  (13 missing) |  | 54.3 (15.8) | 50.0 (18.0) | 60.3 (12.0) |  | 0.029 |
|  |  | **Country of origin** | | |  |  |
|  |  | **The Philippines** | **Indonesia** | **Myanmar/ Sri Lanka** |  |  |
| Preference for management style  (12 missing) |  | 50.3 (17.0) | 57.5 (16.0) | 56.1 (12.2) |  | 0.024 |

* Only significant associations are reported, p<0.05

** The categories ‘Buddhist/Sikh’ and ‘No religion’ were excluded from the test due to low frequencies
